# *VPS4B* mutation impairs the osteogenic differentiation of dental follicle cells derived from a patient with dentin dysplasia type I

**DOI:** 10.1038/s41368-020-00088-z

**Published:** 2020-07-31

**Authors:** Qiang Li, Fangli Lu, Tianxuan Chen, Ke Zhang, Yuping Lu, Xiaocong Li, Yingying Wang, Ling Liu, Qing Tian, Fu Xiong, Dong Chen

**Affiliations:** 1 Department of Stomatology, the First Affiliated Hospital of Zhengzhou University, Zhengzhou, China; 2 The Academy of Medical Sciences, Zhengzhou University, Zhengzhou, China; 3 Department of Stomatology, Henan Provincial Hospital, Zhengzhou, China; 4 Implant Dental Clinic, Zhengzhou, China; 5 Department of Stomatology, Puyang People’s Hospital, Puyang, China; 6 Department of Medical Genetics, School of Basic Medical Sciences, Southern Medical University, Guangzhou, China

**Keywords:** Genetics research, Disease genetics, Dental diseases, Dentistry

## Abstract

A splicing mutation in *VPS4B* can cause dentin dysplasia type I (DD-I), a hereditary autosomal-dominant disorder characterized by rootless teeth, the etiology of which is genetically heterogeneous. In our study, dental follicle cells (DFCs) were isolated and cultured from a patient with DD-I and compared with those from an age-matched, healthy control. In a previous study, this DD-I patient was confirmed to have a loss-of-function splicing mutation in *VPS4B* (IVS7 + 46C > G). The results from this study showed that the isolated DFCs were vimentin-positive and CK14-negative, indicating that the isolated cells were derived from the mesenchyme. DFCs harboring the *VPS4B* mutation had a significantly higher proliferation rate from day 3 to day 8 than control DFCs, indicating that *VPS4B* is involved in cell proliferation. The cells were then replenished with osteogenic medium to investigate how the *VPS4B* mutation affected osteogenic differentiation. Induction of osteogenesis, detected by alizarin red and alkaline phosphatase staining in vitro, was decreased in the DFCs from the DD-I patient compared to the control DFCs. Furthermore, we also found that the *VPS4B* mutation in the DD-I patient downregulated the expression of osteoblast-related genes, such as *ALP*, *BSP*, *OCN, RUNX2*, and their encoded proteins. These outcomes confirmed that the DD-I-associated *VPS4B* mutation could decrease the capacity of DFCs to differentiate during the mineralization process and may also impair physiological root formation and bone remodeling. This might provide valuable insights and implications for exploring the pathological mechanisms underlying DD-I root development.

## Introduction

Dentin dysplasia type I (DD-I, OMIM: 125400) is a rare, autosomal-dominant, heritable, non-syndromic disorder characterized by obliterated pulp chambers, diminutive roots, and severe hypermobility of teeth.^1–4^ This is also often combined with frequent periapical radiolucencies in apparently sound teeth, both in the deciduous and permanent dentitions, with an estimated incidence of 1/100 000.^5–7^ DD-I increases the risk of early tooth loss and causes functional and esthetic disturbances. The initial information on DD-I mainly originated from reports of isolated cases owing to a low incidence. The genetic defects underlying DD-I were subsequently identified by genetic screening in affected families. To date, gene mutations in *SMOC2*, *VPS4B*, and *SSUH2* in three affected families from different countries have been identified, which strongly suggests that this disease is genetically heterogeneous.^4,8–10^ Despite major advancements in knowledge regarding molecular and cellular involvement in DD-I, the pathogenesis of this dysplasia remains undefined.

IVS7 + 46C > G, a splicing mutation that is genetically linked to DD-I in the extended Chinese family of this patient, was identified in the *VPS4B* gene.^10^ This gene is located on chromosome 18q21.33 and encodes a member of the AAA ATPase family.^11^ The VPS4B protein is an important component of the endosomal sorting complexes required for the transport (ESCRT) machinery^12^ and plays crucial roles in multiple cellular processes, including the formation of multivesicular bodies, virus budding,^13^ the abscission of cytokinesis,^14^ and degradation of various membrane receptors.^15^ However, the role of *VPS4B* in the development of other cell types, especially odontogenic cells, remains unclear. In our previous study, we demonstrated that the patient with affected teeth not only had dentin malformations but also had teardrop-shaped lacunae and a decreased organic content in their cementum.^4,16^ Additionally, it has been reported that the affected teeth are supported by insufficient alveolar bone, and the cementum is thin, sparse, or absent.^17,18^ These findings provide important evidence that *VPS4B* may cause imperfect cementogenesis and potentially affect the surrounding alveolar bone during mineralization development.

Interestingly, the dental follicle that originates from cranial neural crest cells, is a loose connective tissue that spherically surrounds the developing tooth germ in the early stages of development.^19–22^ This dental follicle is considered the top candidate for the origin of cementoblasts since it can create cementum-like tissues without epithelial cells in vivo.^23,24^ Dental follicle cells (DFCs) reside in this ectomesenchymally derived, sac-like connective tissue. The normal differentiation of DFCs is essential for cementogenesis as well as surrounding alveolar bone development and formation. Many studies have reported that the differentiation of DFCs is always coordinated with root development.^22,25,26^ Moreover, DFCs are highly considered for the generation of biological tooth roots and for the regeneration of alveolar bone. It has been reported that rat DFCs form a tooth root when seeded on scaffolds of a treated dentin matrix and transplanted into an alveolar fossa microenvironment.^27^ Recent studies have focused on the characteristic and osteogenic differentiation of DFCs for all kinds of diseases, one such example being cleidocranial dysplasia,^28–30^ which is failure of tooth eruption associated with a parathyroid hormone-related peptide.^31^ However, thus far, no data exist on the potential functional roles that DFCs may have during root development in DD-I. DFCs can be obtained from impacted third molars^32,33^ and have been shown to possess the capability of osteogenic differentiation in vitro when induced with the appropriate osteogenic medium.^26,34,35^ Furthermore, the *VPS4B* gene is one of the important contributors to root formation and is widely expressed in human tissues. Therefore, DFCs are a valuable tool to investigate osteogenic differentiation and to explore the mechanisms through which *VPS4B* affects the functions of these cells.

In our present study, we used DFCs as valuable tools to investigate differences in osteogenesis between a healthy individual and a DD-I patient, with the aim of determining the impact of a *VPS4B* mutation on the osteogenesis capacity of DFCs in DD-I, which has not been previously explored. These findings may contribute to the further understanding of the pathological mechanisms underlying DD-I root development.

## Results

### Characterization and growth potential of DFCs in vitro

**Fig. 1:**
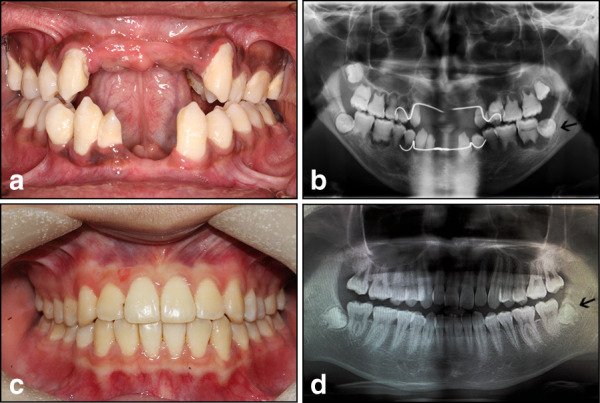
Intraoral images and panoramic radiographs from the individuals. **a** Intraoral image of the patient with DD-I. **b** Panoramic radiograph of the patient with DD-I. **c** Intraoral photo of the healthy control. **d** Panoramic radiograph of the healthy control. The apical development of the third molars of the DD-I patient and the healthy control was not yet completed (black arrow)

During clinical treatment, the DD-I patient with the *VPS4B* mutant had the impacted mandibular wisdom tooth removed. The third molar (at the root developing stage) was extracted and collected from both the DD-I patient and an age-matched healthy adult who underwent orthodontic treatment after informed consent was obtained (Fig. 1).

**Fig. 2:**
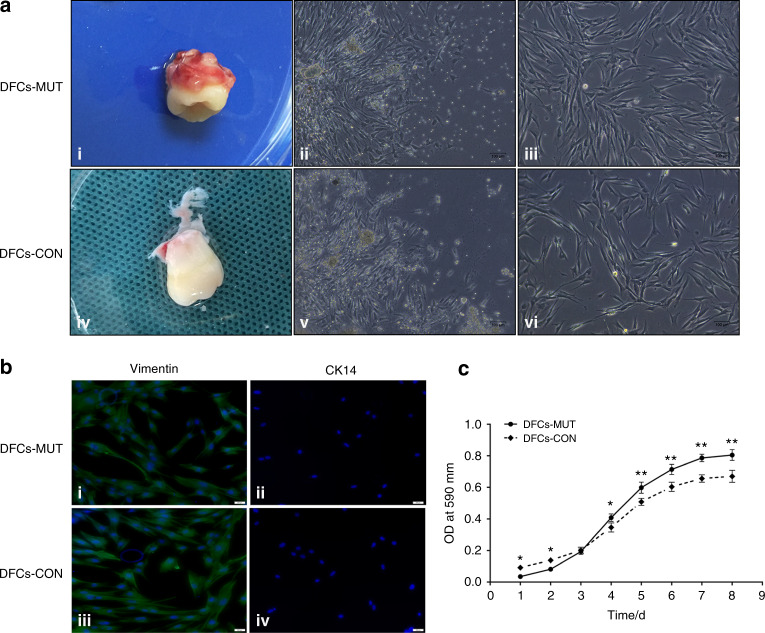
Representative images showing the morphology and growth potential of DFCs from the healthy control and the patient with DD-I in vitro. **a**-i, iv The third molar was harvested from the patient with DD-I and the healthy control. **a**-ii, v The DFCs-MUT and DFCs-CON from the primary passage grew radially and presented with fusiform, spindle, or polygonal morphologies. **a**-iii, vi Representative images showing the morphologies of third-passage DFCs-MUT and DFCs-CON. The DFCs appeared spindle or stellate shaped with a typical fibroblast-like morphology. **b** Identification of isolated DFCs. **b**-i, ii DFCs-MUT were positive for vimentin but negative for CK14. **b**-iii, iv DFCs-CON were also vimentin-positive and CK14-negative. **c** Proliferation curves of DFCs-MUT and DFCs-CON at the indicated time points. Relative cell numbers were determined every day during the one-week experiment. DFCs-MUT, VPS4B mutation in the DFCs from the patient with DD-I, DFCs-CON, DFCs from a normal individual as a control (*P < 0.05 and **P < 0.01)

First, human dental follicles were harvested and subjected to enzymatic digestion to isolate DFCs. Only a small number of single dental follicle tissue cells attached to the plastic surface, and non-adherentcells were removed by changing the medium. We found that the adherent tissues were visible after being cultured for 24 h. Subsequently, a large number of primary passage cells around the tissue mass grew radially for nearly 5 days. At this time point, the cells presented a fusiform, spindle, or polygonal morphology butalso had an excess of epithelial tissue (Fig. 2a–ii, v). From the 7^th^ to 10^th^ days, the cells converged and covered approximately 80% of the bottom of the culture flask. In addition, the cells from passages 3 and 4, which were isolated by plastic adherence, exhibited spindle or stellate shapes and were characterized by a typical fibroblast-like morphology (Fig. 2a–iii, vi).

Vimentin and cytokeratin 14 (CK14) immunofluorescence staining was first performed to verify the source of the cultured cells. The DFCs were vimentin-positive and CK14-negative, indicating that the cells were derived from the mesenchyme (Fig. 2b). The morphology of the cells was not obviously different; however, the subculture time differed between the two groups. In general, DFCs with the *VPS4B* mutation appeared after 3-4 days, while 5-6 days were required for the control DFCs, suggesting that the *VPS4B* mutant cells grew faster. The 3-(4,5-dimethylthiazol-2-yl)-2,5-diphenyltetrazolium bromide (MTT) assay showed that the *VPS4B* mutation significantly increased the proliferation rate of DFCs compared to that of the control cells from day 3 to day 8, indicating that *VPS4B* is involved in cell proliferation (Fig. 2c).

### Osteogenic potential of DFCs in vitro

**Fig. 3:**
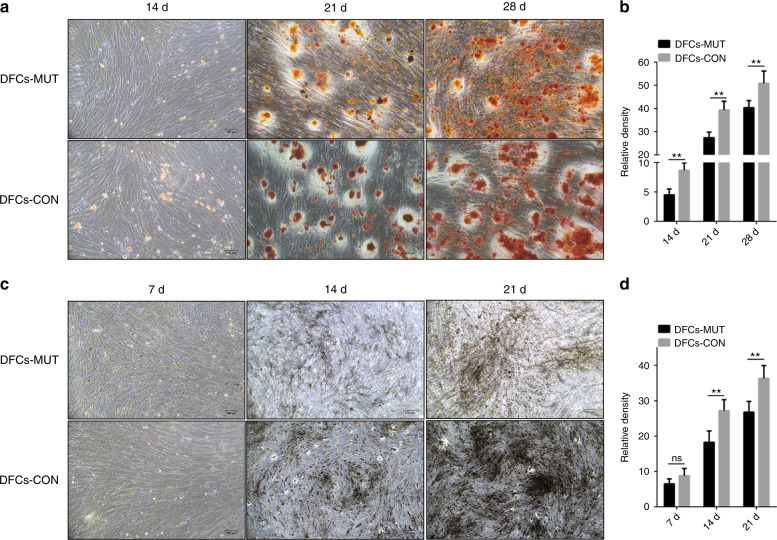
Representative images from the ARS (top) and ALP (bottom) staining of DFCs-MUT and DFCs-CON at the indicated time points after the induction of differentiation, respectively. The data are presented as the means ± SD of three independent experiments (*P < 0.05 and **P < 0.01)

The course of osteoblast differentiation in DFCs was analyzed through the following experiments. The formation of mineralized nodule deposits over a period of 14, 21, and 28 days in culture was confirmed through alizarin red solution (ARS) staining. The intensity of the mineralization was enhanced with the increase in culture time. However, DFCs with the *VPS4B* mutation showed significantly smaller and fewer calcium nodules than the control DFCs at every time point (Fig. 3a, b). ALP staining results showed that the number of positive cells in both cell types increased with increasing mineralized induction time, while there was a lower number of positive cells in the mutated DFCs at each time point (Fig. 3c, d).

### Changes in osteogenic genes and proteins

**Fig. 4:**
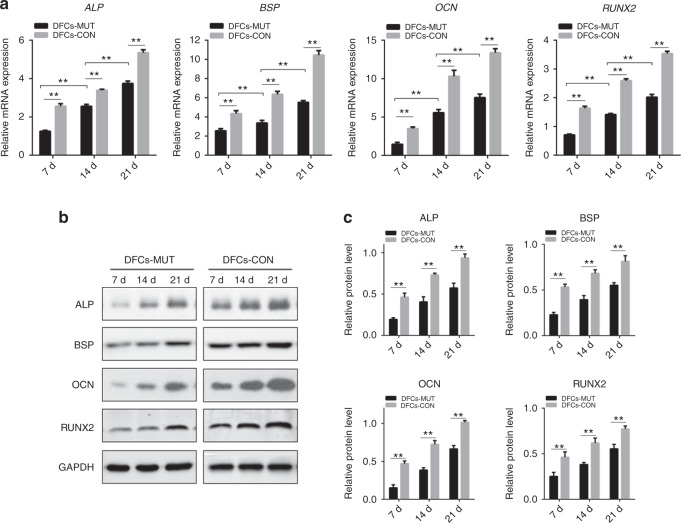
Analysis of the mRNA and protein expression of osteogenic differentiation markers during the osteogenic differentiation of DFCs. **a** Histograms showing the relative mRNA levels of *ALP, BSP, OCN*, and *RUNX2* during the induction of differentiation. The mRNA levels were determined using RT-PCR with GAPDH as a reference for normalization. **b** Representative western blots showing the protein levels of ALP, BSP, OCN, and RUNX2. **c** Histograms showing the relative protein levels of ALP, BSP, OCN, and RUNX2 during the induction of differentiation. Values are means ± SD of three independent experiments (*P < 0.05 and **P < 0.01)

To determine whether the *VPS4B* mutation affected DFCs differentiation, we analyzed the changes in the levels of osteogenic-specific mRNA and protein markers in induced DFCs using real-time polymerase chain reaction (RT-PCR) and western blotting, respectively. We isolated total RNA to compare gene expression in DFCs after 0, 7, 14, and 21 days of mineralization induction in both groups (both at passage 3). We found notable differences in the gene expression of *ALP*, *OCN*, *BSP*, and *RUNX2*. The gene expression levels of *ALP*, *OCN*, *BSP*, and *RUNX2* increased gradually with increasing osteogenic induction time in both groups. However, the expression of each gene in DFCs with the *VPS4B* mutation was significantly lower than that in control DFCs at each time point (Fig. 4a).

Western blotting showed that the osteogenic-specific proteins ALP, OCN, BSP, and RUNX2 were expressed from day 7, and the expression levels of these proteins increased gradually in the two groups. The protein expression in DD-I DFCs was significantly downregulated compared with that in the control DFCs at every time point (Fig. 4b, c).

## Discussion

DD-I is a rare, autosomal-dominant dental dysplasia disorder affecting both deciduous and permanent dentition, with an unclear etiology. It was first reported as “rootless teeth” in 1922 by Ballschmiede, who found a family with six children manifesting with short, blunted roots and premature tooth loss.^36^ The estimated incidence of DD-I is 1/100,000,^3,7,37^ and thus, knowledge of this disorder mainly originates from reports of isolated cases. Individuals with DD-I frequently present with tooth hypermobility combined with diminutive roots, aberrant dentin formation, obliterated pulp chambers, and numerous periapical radiolucencies in non-carious teeth.^2,7,16^ Therefore, the condition currently remains a clinical management dilemma and often leads to challenges in genetic counseling.

Interestingly, various studies have reported the existence of stem cells and precursor cells in the dental follicles of humans, rats, and other species,^20,21,26,38^ which possess multi-lineage differentiation potential for cementoblasts, periodontal ligament cells, and osteoblasts. DFCs are known to have important functions during the formation of the cementum and root-surrounding alveolar bone. The osteogenic abilities of DFCs are crucial for this physiological process. Presently, most studies have focused on the characteristic and osteogenic differentiation of DFCs in different kinds of diseases, such as cleidocranial dysplasia,^28–30^ which is the failure of tooth eruption associated with parathyroid hormone-related peptide.^31^ However, no studies have compared DFCs between patients with DD-I and healthy individuals. Thus, to gain a better understanding of this disease, we explored the relationship between a *VPS4B* mutation and the osteogenesis mineralization abilities of DFCs. We designed this study to test the hypothesis that the *VPS4B* mutation affects osteogenic differentiation in DFCs, thereby disrupting the modulatory effects of DFCs on the differentiation of osteoblasts and consequently interfering with root and surrounding hard tissue formation and remodeling. In this study, we compared the proliferation and induced osteogenesis abilities of DFCs from a patient with DD-I with those from a healthy control.

Until now, few studies have compared cell proliferation between patients with DD-I and healthy individuals. One study^39^ performed proliferation assays using Cell Counting Kit-8, which indicated that human dental pulp stem cells (hDPSCs) transduced with an shRNA-Vps4b lentivirus proliferated slower than their counterparts transduced with a control lentivirus over 7 days. However, in our study, the DD-I DFCs growth rates were significantly increased compared to the control DFCs growth rates after 3 days of culture. The different proliferative capacity between hDPSCs and DFCs in DD-I may be related to the different sources of these cells. Further studies may be needed to better understand the mechanism underlying the effects of the *VPS4B* mutation on cell proliferation.

In this study, we examined the role of *VPS4B* in DFCs osteogenic differentiation. To this end, we performed ARS and ALP staining to monitor mineralization, and we examined the expression levels of the osteogenic differentiation markers *ALP*, *BSP*, *OCN* and *RUNX2*. ARS and ALP staining provided further evidence that *VPS4B* is involved in the osteogenic differentiation of DFCs and therefore may regulate root formation and bone remodeling during development. Consistent with the data from other studies,^34,40^ our results show that DFCs cultured in osteogenic induction medium increased the expression of these osteoblast-specific markers. In this study, we analyzed the mRNA expression of these four factors, and significant variations were found between DD-I DFCs and control DFCs. Western blotting analysis also demonstrated the same trend in DFCs between the two subjects. According to the above results, we confirmed that the *VPS4B* mutation could increase the proliferation rate and significantly downregulate the expression of osteoblast-related genes and proteins in vitro, indicating a decreased osteogenic capacity in the patient with DD-I due to the *VPS4B* mutation.

In our study, the detailed molecular mechanisms underlying the functions of *VPS4B* during DFCs proliferation and osteogenic differentiation have yet to be fully elucidated. Notably, Wnt/β-catenin signaling is functionally significant for root odontogenesis and cementogenesis.^41–43^ One study showed that *VPS4B* acts as a regulator of hDPSC proliferation and differentiation via Wnt-β-catenin signaling.^39^ Moreover, it has been demonstrated that the overexpression or knockdown of *VPS4B* in human gingival fibroblasts markedly increased or decreased the expression of CHMP4B, Wnt5a, and β-catenin. The *VPS4B*-CHMP4B-Wnt5a-β-catenin regulatory pathway might regulate odontoblast differentiation and root formation.^10^ Thus, we speculated that *VPS4B* might regulate the osteogenic differentiation of DFCs through Wnt/β-catenin signaling, subsequently influencing root formation and bone remodeling. Future studies that test whether *VPS4B* affects other molecules or factors in the Wnt or other signaling pathways will undoubtedly improve our understanding of the molecular mechanisms underlying DD-I root development.

In addition, this study is a single-patient study with certain limitations, and the patient’s mutation of *VPS4B* may be different from variants of this gene in other patients with DD-I. Studies in more patients with DD-I and an individualized analysis of their characteristics could be performed to investigate this disorder further in the future. This would allow researchers to develop a more comprehensive understanding of this disorder with stronger evidence.

## Material and methods

### Cell isolation and culture

This was an experimental study. The study protocol and patient consent were reviewed and approved by the Medical Ethics Committee of the First Affiliated Hospital of Zhengzhou University, China (2019-KY-104). The homolateral mandibular third molars, which had an immature root apex, were extracted and collected from both the DD-I patient and the age-matched healthy adult who underwent orthodontic treatment after informed consent was obtained. The attached dental follicles were gently separated from the tooth surface, and the dental papilla tissue was discarded. The surfaces of the tissues were thoroughly washed with phosphate-buffered saline (PBS; HyClone, China) and minced using a sterilized scalpel. The minced tissues were digested in a solution of 1 mg/ml collagenase type I (Bioleaf, Shanghai, China) for 1 h at 37 °C to obtain a cell suspension. The supernatant was discarded after the cell suspension was centrifuged at 12 000 r·min^−1^ for 5 min. Thereafter, dental follicle explants were seeded into T-25 culture flasks, which contained 2 ml of an alpha modification of Eagle’s medium (α-MEM; HyClone, China), 10% heat inactivated fetal bovine serum (FBS; Gibco, Grand Island, NY, USA), and 1% penicillin/streptomycin (HyClone, UT, USA); the cells were incubated at 37 °C and 5% CO_2_ in a humidified atmosphere. After 24 h, the medium was replaced with fresh 10% α-MEM. After cells had adhered to the plastic dish surface, non-adherent cells were removed by changing the medium after overnight incubation. Thereafter, the culture medium was changed once every 3 days, and cell morphology images of each group were captured via a phase-contrast inverted microscope (Sunny, Ningbo, China). At 80% confluency, cells were harvested with 0.25% trypsin and 0.02% EDTA (Dingguo Biotechnology, Beijing, China) and passaged in α-MEM containing 10% FBS for subsequent experiments. Cells from passages 2 to 5 were used for the experiments in this study.

For induction of in vitro osteogenic differentiation, cells that were digested with 0.25% trypsin were grown on 6-well plates at a density of 2 × 10^4^ cells per well in 10% α-MEM. At 80% confluency, cells were replenished with osteogenic medium consisting of α-MEM with 10% FBS, 1% penicillin/streptomycin, 10^–8^ mol·L^−1^ dexamethasone, 50 μg·mL^−1^ ascorbic acid, and 10 mmol·L^−1^ β-glycerophosphate (Sigma-Aldrich, St. Louis, MO, USA). The cells were then cultured for a period of 28 days, and the medium was then changed every 3 days.

### Identification of isolated cells

The purified DFCs were further identified using vimentin and CK14 immunofluorescence staining. A single-cell suspension was formed by digesting the cells with 0.25% trypsin and centrifuging them for 5 min at 12 000 r·min^−1^. The cells were plated on coverslips at a density of 2 × 10^4^ cells per well in a 24-well plate. At 70%–80% confluency, cells were fixed with 4% paraformaldehyde (PFA; Dingguo Biotechnology) for 20 min. After being washed in PBS three times for 5 min, the cells were permeabilized with 0.25% Triton X-100 (Sigma-Aldrich) at 37 °C for 15 min. After being washed in PBS, cells were blocked with 1% normal goat serum (ZSGB-BIO, Beijing, China) at 37 °C for 30 min and incubated overnight with rabbit anti-vimentin (Gibco) and rabbit anti-CK14 (Gibco) at 4 °C. After another PBS wash, the cells were incubated with goat anti-rabbit secondary antibodies (ZSGB-BIO) at 37 °C for 1 h. Following another PBS wash, 4′,6-diamidino-2-phenylindole (DAPI; Invitrogen, NY, USA) was used to stain cell nuclei at 37 °C for 3 min; staining images were captured using a fluorescence microscope (Nikon, Tokyo, Japan).

### Evaluation of cell proliferation

Growth profiles of cells cultured between days 1 to 8 from the two groups were measured by an MTT assay. Briefly, cells at passage 3 were seeded at a density of 1 000 cells per well in 96-well plates containing 10% α-MEM. At each time point, 20 μL of MTT solution (1 mg·mL^−1^; Sigma-Aldrich) was added to each well; thereafter, all the plates were returned to standard tissue incubator conditions for an additional 4 h. After the removal of the MTT solution, 150 μL dimethyl sulfoxide (DMSO; Sigma-Aldrich) was added to each well and incubated while shaking in the dark for 15 min. The cell proliferation profiles were determined from the measurements obtained at a wavelength of 490 nm with a microplate reader (Shanghai Precision Instrument Co. Ltd., Shanghai, China). Experiments for all samples were repeated in triplicate.

### Alizarin red staining

After 14, 21, and 28 days of induction, the presence of mineralized depositions was determined by staining with alizarin red. The cells were rinsed with PBS and fixed with 70% ice-cold ethanol. After being washed with distilled water, the cells were treated with ARS (pH 4.1, Sigma-Aldrich) at room temperature for 30 min. Then, the excess dye was removed, and the cells were rinsed with distilled water until the background became clear. Calcium depositions could be visualized by their intense red color. All images were captured at the same magnification and light intensity. Experiments on all samples were performed in triplicate. The relative intensities were analyzed and compared to their respective controls using ImageJ software (NIH, USA).

### Alkaline phosphatase staining

Alkaline phosphatase staining was carried out as described previously.^44^ The cells were seeded in 6-well plates at a density of 2 × 10^4^ cells per well. After in vitro osteogenic induction for 7, 14, and 21 days performed as previously described, alkaline phosphatase staining was carried out. Briefly, culture wells were rinsed three times with PBS, fixed in 95% alcohol for 1 min, washed three times with PBS, incubated with the substrate mixture (2% sodium β-glycerophosphate, 25 ml; 2% sodium barbiturate, 25 ml; distilled water, 50 ml; 2% calcium chloride, 5 ml; 2% magnesium sulfate, 2 ml; and several drops of chloroform; Solarbio, Beijing, China) at 37 °C for 2 h, washed twice with pure water, incubated in 2% cobalt nitrate (Solarbio) for 2 min, and finally incubated with 2% amine sulfide (Solarbio) for 2 min. Stained cell images were captured at the same magnification and light intensity.

### Real-time PCR analysis

Total RNA from cells was harvested after 0, 7, 14, and 21 days of osteogenesis induction using TRIzol Reagent (Invitrogen) and subjected to reverse transcription using the cDNA Reverse Transcription Kit (Takara, Dalian, China) in separate groups. RT-PCR was performed using SYBR Premix DimerEraser (Takara) on a LightCycler 480 (Roche, Indianapolis, IN, USA). The sequences of the related gene primers for RT-PCR are presented below (Table 1). The 2^−∆∆Ct^ method was used to calculate the relative mRNA expression of each target gene with *GAPDH* as the reference. All samples were assayed in triplicate.

**Table 1:** Sequences of the primers used for the real-time PCR

Primers	Abbreviation	Sequences (5′ to 3′)
*Alkaline phosphatase*	*ALP*	forward: GGACCATTCCCACGTCTTCAC
		reverse: CCTTGTAGCCAGGCCCATTG
*Osteocalcin*	*OCN*	forward: CCCAGGCGCTACCTGTATCAA
		reverse: GGTCAGCCAACTCGTCACAGTC
*Bone sialoprotein*	*BSP*	forward: CTGGCACAGGGTATACAGGGTTAG
		reverse: ACTGGTGCCGTTTATGCCTTG
*Runt-related transcription factor 2*	*RUNX2*	forward: GAGGGCACAAGTTCTATCTG
		reverse: GGTGGTCCGCGATGATCT
*Glyceraldehyde 3-phosphate dehydrogenase*	*GAPDH*	forward: GCACCGTCAAGGCTGAGAAC
		reverse: TGGTGAAGACGCCAGTGGA

### Western blot analysis

Western blotting was carried out as described previously.^45^ Total protein was extracted from the cultured cells using RIPA lysis buffer (Beyotime, Nanjing, China), which was supplemented with a protease inhibitor cocktail according to the manufacturer’s instructions. The protein mixtures were centrifuged to remove cellular debris. The extracted proteins were separated by 10% sodium dodecyl sulfate-polyacrylamide gel electrophoresis (SDS-PAGE) and electrotransferred to polyvinylidene fluoride (PVDF) membranes (Amersham, Little Chalfont, UK). The PVDF membranes were blocked for 2 h at room temperature using 5% non-fat milk (BD Biosciences, San Jose, CA). The membranes were incubated at 4 °C overnight with the following primary antibodies: anti-OCN (1:1 000; Boster Biological Technology, Wuhan, China), anti-BSP (1:1 000; Boster Biological Technology), anti-ALP (1:1000; Proteintech Group, Wuhan, China), anti-RUNX2 (1:1 000; Proteintech Group), and anti-GAPDH (1:1 000; Boster Biological Technology). Subsequently, the membranes were incubated with horseradish peroxidase (HRP)-conjugated secondary antibodies (ZSGB-Bio) for 1 h at room temperature. The immunoreactive bands were visualized using an ECL chemiluminescence reagent (Solarbio). The relative intensities were analyzed and compared to their respective controls using ImageJ software (NIH, USA).

### Statistical analysis

All results were obtained from triplicate samples. Data are presented as the means ± standard deviation and compared by Student’s *t*-test or one-way analysis of variance using the SPSS 21.0 software package (IBM Corporation, Armonk, NY, USA). *P*-values < 0.05 were considered to be statistically significant.

## Conclusion

In summary, the present results confirmed that the DD-I-associated *VPS4B* mutation affected the osteogenic differentiation of DFCs and therefore may regulate root formation and bone remodeling during development. These findings provide valuable insights and implications for rootless teeth in patients with DD-I. Further studies are still needed to determine how *VPS4B* regulates the factors related to root formation and bone remodeling, as well as the intrinsic mechanism of these clinical manifestations in DD-I patients.
